# Educating to sexuality care: the nurse educator’s experience in a multicenter study

**DOI:** 10.3389/fpsyg.2023.1206323

**Published:** 2023-07-24

**Authors:** Cinzia Gradellini, Daniela Mecugni, Elena Castagnaro, Fátima Frade, Maria da Luz Ferreira Barros, Sara Palma, María Jesús Bocos-Reglero, Sagrario Gomez-Cantarino

**Affiliations:** ^1^Reggio Emilia Nursing Course, University of Modena and Reggio Emilia, Modena, Italy; ^2^Department of Business Economics, Health and Social Care, University of Applied Sciences and Arts of Southern Switzerland, Manno, Switzerland; ^3^Azienda Unitaà Sanitaria Locale – IRCCS, Reggio Emilia, Italy; ^4^Departamento de Enfermagem da Criança e do Jovem, Escola Superior de Enfermagem de Lisboa, Lisboa, Portugal; ^5^Nursing Research, Innovation and Development Centre of Lisbon (CIDNUR), Nursing School of Lisbon, Lisbon, Portugal; ^6^Centre for Public Administration and Public Policies, Institute of Social and Political Sciences, Universidade de Lisboa, Lisbon, Portugal; ^7^Department of Nursing, University of Évora, Évora, Portugal; ^8^Comprehensive Health Research Center (CHRC), Évora, Portugal; ^9^School of Health, Polytechnic of Santarém, Santarém, Portugal; ^10^Faculty of Physiotherapy and Nursing, University of Castilla-La Mancha, Toledo, Spain; ^11^Health Sciences Research Unit: Nursing (UICISA: E), Nursing School of Coimbra (ESEnfC), Coimbra, Portugal

**Keywords:** sex education, health education, sexuality, faculty nursing, health knowledge, attitudes, practice

## Abstract

**Background:**

Sexuality is an issue inherent in the lives of all human beings. Education for Sexuality takes place informally, through relationships with the environment, with the family as a model, and formally, as a pedagogical practice in Teaching. Education for sexuality is recognized as an instrument of social transformation that leads to changes in behaviors and norms related to sexuality.

**Objectives:**

Knowing the perception of nursing professors about sexuality education in professional training, recognizing attitudes of these professors in relation to sexual education and identifying barriers in education for sexuality.

**Methods:**

Exploratory and descriptive study, using qualitative methodology. Data collection was carried out from semi-structured interviews and thematic analysis.

**Results:**

The interviewees consider sexuality education to be very important, being taught in the nursing course, addressing different themes. In general, they reported feeling comfortable teaching these topics. The identified barriers to the level of education students are in, students’ knowledge and reactions to the topic, religious and cultural issues, and the time available to talk about the topic and professional aspects.

**Conclusion:**

Sexuality is a fundamental theme in nursing education and needs to be further explored to overcome the barriers associated with its approach.

## 1. Introduction

In 2017, the World Health Organization (WHO), defined several terms related to sexual and reproductive health. These terms include sex, sexual health, sexuality, and sexual rights. Going deeply into the definition of sexual health, it is described as: “a state of physical, emotional, mental, and social well-being about sexuality; it is not merely the absence of disease, dysfunction, or infirmity. (…) it requires a positive and respectful approach to sexuality and sexual relationships, as well as the possibility of having pleasurable and safe sexual experiences, free of coercion, discrimination, and violence. For sexual health to be attained and maintained, the sexual rights of all persons must be respected, protected, and fulfilled” [World Health Organization (WHO), n.d.].

Sexuality education promotes the sexual rights of all people, is supported by the Sustainable Development Goals (SDGs), namely by SDG 3, which promotes Gender Equality, and SDG 4, which ensures Quality Education for all by lifelong. The results of this article, which is part of the European Project Sexuality Education: A Breakthrough for European Health (EdSeX), meet the priorities of SDG 3, linked to the objective of eliminating all forms of violence against women and girls, eliminating harmful practices, such as child marriage and female genital mutilation, ensuring universal access to sexual and reproductive health and rights, and increasing the use of information and communication technologies, to promote women’s empowerment; and SDG 4, which focuses on ensuring quality education for all throughout life, helping societies to deal with problems related to health risks and health promotion (Vilaça, 2017; United Nations, 2030).

Through sexuality, people express their most intimate feelings of individuality and their need for emotional closeness with other human beings. Sexuality is not just about sexual intercourse, it is about the concept of people as men and women, about their manliness or femininity (Coleman et al., 2013).

To maximize the value of sexuality and sexual health education, it is crucial to understand how to optimize comfort in the delivery and reception of this education. Sexual health education aims to impart young people with the information they need, to make informed decisions about their sexual health (Mueller et al., 2008) and provides students with the knowledge and skills necessary to understand sexual development, establish healthy relationships, prevent sexually transmitted diseases, and unintended pregnancy, becoming healthy adults (Buston et al., 2002). Sexual health education has been shown to delay the initiation of sexual intercourse, reduce sexual activity and the number of sexual partners, increase condom or contraceptive use, and improve academic achievement (Kirby et al., 2007).

Previous studies show that several factors could impact teachers’ delivery of health education (Vamos and Zhou, 2003) and, more specifically, their delivery of sexual health education (Eisenberg et al., 2012). Comfort has been identified as one of those factors (Cohen et al., 2012). In a study conducted by Rose et al. (2018), interviews with middle school health education teachers and focus groups with students were collected to examine the factors that influence the perceived comfort of those who deliver and receive education in a public school district. Findings identified key barriers including disruptive behavior, insufficient time, and lack of dedicated classrooms. Some key facilitators to comfort included professional development and establishing ground rules (Perez et al., 2013; Rose et al., 2018).

Barriers can be substantial for teachers, and some researchers have explored ways to incorporate online approaches and computer-assisted instruction into sexual health education to enhance student learning experiences and minimize some of these barriers (Roberto et al., 2007; Chen and Barrington, 2017). Although sexual health education incorporating online platforms has demonstrated some success in shifting students’ sexual health knowledge and attitudes (Evans et al., 2000), such approaches are best utilized as a part of a comprehensive instructional design that uses multiple delivery systems, both teacher and student led.

Rose et al.’s (2018) study highlighted facilitators that may help increase comfort levels in the classroom, including the establishment of ground rules, using a question box, and offering professional development opportunities for teachers to improve classroom management. Creating a comfortable, safe, and supportive environment is essential, and this type of environment has been identified by researchers as a key characteristic of effective sexual health education curricula (Kirby et al., 2006; Rose et al., 2018).

Sexual health education works best in classrooms where there is mutual trust and comfort for both teachers and students (Mkumbo, 2012). Comfort has been expressed in the context of teachers’ knowledge about sexual health, as well as their comfort in teaching and discussing sexual health topics (Cohen et al., 2012; Mkumbo, 2012). The teachers’ comfort has also been associated with their coverage of sexual health topics and their ability to address student reactions to sexual health content, as well as other classroom management issues. The understanding of the vulnerabilities of university students is fundamental for the development of training courses that create environments for debate and democratic participation with access not only to information, but effective training on sexuality, which leads to practices reflected and internalized by the student in their daily lives (Ninomiya, 2010; Mkumbo, 2012; Brancaleoni et al., 2018).

Research studies in health have reported many nurses are not comfortable speaking with their patients about topics related to sexual health. Many nurses tend to neglect sexual health care because they do not feel they have sufficient education, experience, or confidence to properly engage with patients (Sung et al., 2016; Martel et al., 2017), and this creates a barrier to the patient’s sexual care (Klaeson et al., 2017).

Several researchers have published about patients’ benefits when nurses had comprehensive training on topics related to sexual health care (Sung et al., 2016; Yingling et al., 2017). Furthermore, nurses who were formally trained on how to best deliver sexual health information were more effective in addressing patients’ sexual health concerns proactively instead of reactively (Sung et al., 2016).

Three studies highlighted barriers experienced by nurses as the lack of policies about the role of nurses in discussing sexual health care with patients, in addition to organizational and management support to allow nurses the time to engage with patients about their sexual health (Moore et al., 2013; Krouwel et al., 2015; Jonsdottir et al., 2016). This lack of support lead to role confusion decreased comfort and the belief sexuality is not an essential aspect of the nursing assessment (Beebe et al., 2021).

Time constraints and privacy concerns have been expressed as barriers in many of the research studies. Attitudes and beliefs of nurses working in oncology, ranged from the belief that patients would feel embarrassed or offended, sexual health is a difficult issue to address, a lack of privacy, and/or there is not enough time to discuss sexual health (Moore et al., 2013; Krouwel et al., 2015; Jonsdottir et al., 2016). Huang et al. (2013) noted sexual health care “*can be highly problematic within primary care because of its sensitivity, complexity and the constraints to time and expertise of healthcare professionals*” (Huang et al., 2013). Klaeson et al. (2017) suggested time, lack of education, and nursing profession regulations were contributing factors that led to a barrier to delivering sexual health information to patients (Klaeson et al., 2017).

In several studies, it has emerged that most medical students and medical health professionals do not receive sufficient education on sexual health and do not feel comfortable when dealing with sexual problems. It has been suggested that more sexual health courses are needed in medical schools to overcome this deficiency (Coleman et al., 2013; Turner et al., 2016; Beebe et al., 2021).

Since sexuality education in the area of nursing has an impact on the training of nursing students and on the care they can provide as a nurse in the area of sexuality, this study is important because it identifies the knowledge, attitudes and barriers that nurse educators report when they educate about sexuality, which can influence training and care in this area.

The objective of this study is knowing the perception of nursing professors about sexuality education in professional training, recognize the attitudes of professors in relation to sexual education in nursing education and to identify the barriers in education for sexuality in the nursing degree.

## 2. Materials and methods

### 2.1. Design type

This is an exploratory, descriptive study with a qualitative approach.

### 2.2. Data collection

Data were collected through semi-structured interviews, being the same carried out by the researchers themselves, in the period of August and September 2022. In each country, two interviewers were selected (a total of 8 interviewers), who are part of the European Project Sexuality Education: A Breakthrough for European Health (EdSeX), having contributed to the preparation of the interview guide and were trained to carry it out.

The interview guide questions have been created considering the study of Rose et al. (2018), integrating questions that meet the objectives of the study. It is organized into five parts, the first corresponding to the sociodemographic data to the interviewee, the second to professional data, the third to the perception of the importance of sexual education, the fourth to attitudes/comfort in matters of sexual health and the fifth to pedagogical barriers to sexual health.

The interview guide questions were translated into the language of the partner countries (Spanish, Italian and Portuguese) to facilitate its use, and submitted to a pre-test with teachers with similar characteristics to the research sample, but not involved in the research.

The interviews were carried out in an atmosphere that is pleasant and close to the participant, defined with them to favor communication, had the duration approximately 1 h and were registered by means of recording, after the participant’s authorization.

### 2.3. Sample

International convenience sample composed of 45 teachers of nursing and nurses.

The sample was determined by saturation of the information collected totaling 45 respondents, identified as ES_1 to ES_12 (interviewees in Spain), IT_1 to IT_10 (interviewees in Italy), PTE_1 to PTE_12 (interviewees in Évora, Portugal), PTS_1 to PTS_11 (interviewees in Santarém, Portugal).

Inclusion criteria are: (1) having at least 2 years of experience as a nursing teacher in the field of nursing, (2) mastering the language of the location where the data are collected, and (3) teaching more than 3 h per week. These inclusion criteria ensured a minimum of professional experience in the field of nursing, thus allowing contact with this theme both in theoretical teaching and in clinical practice. Teaching for a minimum of 3 h per week allows nurses who work in clinical practice and do not have teaching experience to be excluded from the sample. Language is important to ensure the absence of language barriers during the interview.

### 2.4. Data analysis

In the data analysis, the Thematic Analysis (TA) method was used to analyze the content of the interviews. We used this method to describe in a detailed and differentiated way the theme of Education for Sexuality in Higher Education in Nursing, allowing us to identify standardized meaning as the main themes emerging from the interviews.

The interviews were analyzed following 6 sequential phases that can be combined, based on the data that emerged:

1. Familiarization with the data: consisting of data reading and re-reading, to become familiar with the contents;
2. Coding: with the identification of labels identifying relevant elements which could be linked to the research outcomes;
3. Generating initial themes: it is based on analysis of previous labels/codes, to identify potential themes;
4. Reviewing themes: to understand if they support a convincing interpretation of the data, answering the research question;
5. Defining and naming themes: developing themes in a single story/interpretation, and giving a name to each of them;
6. Writing up: describe the emerged results, of the existing literature (Braun and Clarke, 2019).
  To guarantee the criteria of reliability and validity of the research, all precepts were followed to guarantee the accuracy of the registration of the data obtained (recording and transcription); verification of the data by four different teams and then the analysis and development of relationships between the data found in the interviews, to ensure consistency between the theoretical constructs and the analysis developed.

### 2.5. Ethical considerations

The ethical principles set out in the Helsinki Declaration have been followed. The permission of the participants was obtained through an informed consent in which they expressed their voluntary desire to participate in the study. Data were recorded anonymously and treated confidentially. The study was authorized by the Research Ethics of University Castilla-La Mancha (UCLM).

## 3. Results

### 3.1. Sociodemographic data

The sample consists of 45 nursing professors and nurses, 26.7% of whom were interviewed by researchers from the University of Castilla-La Mancha (Spain), 26.7% by researchers from the University of Évora (Portugal), 24.4% of the sample was interviewed by researchers from the University of Santarém (Portugal) and 22.2% of the sample comes from the University of Modena and Reggio Emilia (Italy). Globally, the most represented age group is between 40 and 50 years old (49%), followed by the age group from 31 to 40 years old (31%), those over 51 years old (13%) and respondents aged between 20 and 30 years old (7%). The predominant gender is female, representing 62.2% of the sample. In terms of qualifications, 35.6% have a master’s degree (2° cycle) and 35.6% have a Doctorate. This is followed by the Master with 7 professors (15%) and the degree (cycle I) with 6 teachers (13%). Most of the sample works at the University (56%), 13 respondents work at the hospital (29%), 3 work in both contexts (7%), 2 work in the community (Community Nursing) (4%) and 2 do not specify (4%) (Table 1).

**Table 1:** Sociodemographic characteristics (*n* = 45).

Variables		*N*	%
Interviews	University Castilla La Mancha – Spain	12	26.7%
	University of Évora – Portugal	12	26.7%
	University of Santarém – Portugal	11	24.4%
	University of Modena and Reggio Emilia – Italy	10	22.2%
Age	20–30	3	6.7%
	31–40	14	31.1%
	40–50	22	48.9%
	>51	6	13.3%
Gender	Male	17	37.8%
	Female	28	62.2%
Qualification	Bachelor Degree (1° cicle)	6	13.3%
	Master	7	15.6%
	Master Degree (2° cicle)	16	35.6%
	PHD	16	35.6%
Workplace	University	25	55.6%
	Hospital/University	3	6.7%
	Hospital	13	28.9%
	Community nursing	2	4.4%
	Other	2	4.4%
	Total	45	100.0%

The comprehensive reading of the content of the interviews allowed the construction of the following themes: (1) Importance of education for sexuality; (2) Integration in the Study Plan; (3) Content; (4) Barriers; (5) Teachers’ attitudes; (6) Teacher training (Table 2).

**Table 2:** Thematic analysis.

Theme	Sub-theme	Register unit
Importance of education for sexuality	(1) Is important	IT_1, IT_2, IT_3, IT_4, IT_5, IT_6, IT_7, IT_8, IT_9, IT_10, PTS_1, PTS_3, PTS_5, PTS_10, PTS_11, PTE_1, PTE_2, PTE_3, PTE_4, PTE_5, PTE_6, PTE_7, PTE_8, PTE_9, PTE_10, PTE_11, ES2, ES3, ES_7, ES_12
Integration in the Study Plan	(1) Is part of the study plan (2) Isn’t part of the study plan	IT_1, ES_6, ES_9, PTE_3, PTS_3 PTE_6, PTE_9, IT_4
Contents	(1) Gender issues; (2) Ethical dimension of sexuality; (3) Contraceptive methods and sexually transmitted infections; (4) Violence; (5) Emotions and affections; (6) Body Image and female genital mutilation.	IT_1, IT_3, PTE_4, PTE_5, PTS_10 IT_3, PTE_5, IT2, PTS_10, PTS_2 PTS_4, PTS_9, IT10, ES4, IT_1, PTE_4, PTE_5 PTE_3, PTE_4, PTS_10 IT_3, IT_4, IT_6, IT_7, PTE_4, PTS_1, PTS_4,
Barriers	(1) Level of education (2) Taboo subject (3) Students’ knowledge and reactions (4) Cultural and religious issues (5) Lack of time to address sexuality education (6) Professional aspects	IT_3, IT_4, PTE_6, PTS_3, ES_5, PTS_11, PTS_9, PTS_4 IT_1, IT_5, ES_1, ES_6, PTS_3, PTS_4, PTS_8, PTS_9, PTE_3 IT_1, IT_2, IT_3, PTE_1, PTE_2, PTE_4, PTE_5, PTE_6, PTS_1, PTS_8, PTS_9, PTS_11, IT_3, PTS_3, PTE_5 IT_1, IT_3, IT_7, IT_9, IT_10, ES_2; ES_4, PTS_1, PTS_2, PTS_3, PTE_ 3, PTE_8 PTS_2, PTE_1, IT_2, PTE_5, PTS_11,
Teacher’s attitude	(1) Feeling comfortable (2) Discomfort	IT_9, IT_10, ES_5, ES_6, ES_7, ES_8, ES_9, PTE_3, PTE_4, PTE_6, PTE_7, PTE_11, PTE_12, PTS_1, PTS_2, PTS_3, PTS_6, PTS_7, PTS_8, PTS_9, PTS_10, IT_5, ES_2, ES_4, ES_10, ES_11, ES_12, IT_7, PTE_8, PTE_9, PTS_2 IT_1, IT_2, PTS_6, IT_4
Teacher training	(1) Comparison between colleagues (2) Specific training or expertise (3) Importance of continuous updating of knowledge, both subject-specific and pedagogical strategies	IT_2, PTS_4 IT_2, IT_3, IT_4, IT_5, IT_6, ES_1, ES_2, ES_3, ES_4, ES_5, ES_6, ES_7, ES_8, ES_9, ES_10, ES_11, ES_12, PTE_2, PTE_7, PTS_3, PTS_5, PTS_6, PTS_7, PTS_9, PTS_10 IT_5, IT_9, PTS_9, PTS_11, ES_5, ES_12, PTE_8

### 3.2. Importance of education for sexuality

All interviewees agree on the importance of the topic: “*it is a holistic approach to the person, (...) an identity component, essential*” (IT_3); “*it is important because it is a dynamic aspect of health in general*” (PTS_8); “*It is not only important for health professionals, but also for all areas (...) that must consider these aspects because they are relevant for the construction of personality and socially balanced individuals”* (PTE_1). Not only that, a teacher at the University of Santarem reports: “*It is very important to address this issue of sexual health, especially in the area that is maternal and obstetrics health (…) Every health professional should know how to deal with these issues very well*” (PTS_2); “*It is very important since the patient is a biopsychosocial being”* (ES_2, ES_4, ES_7; ES_12).

### 3.3. Integration in the study plan

Sexuality education is part of the study plan of the nursing degree course, in some universities and others where it is not. Which allows us to say that within the theme, integration in the study plan we have the sub-theme, integrates the study plan, and does not integrate the study plan.

When integrated into the study plan for the curriculum, it can be discussed across many different courses, and can use theoretical models of nursing, such as Nancy Roper’s model that describes the activity of life - sexuality, or other specific curricular units, such as Child Health Nursing and Pediatrics, Maternal and obstetric health nursing, Elderly Health Nursing, and psychology. “*This kind of topic is addressed”* (IT_1; ES_6; ES_9); “*The issue of sexual education is part of the curriculum, when we address human needs, we have Nancy Roper’s Theoretical Model as a reference, which addresses the life activity Expressing Sexuality and there the issue of sexuality is addressed. This issue can be addressed in all curricular units, since it is recommended to address all activities of life, according to this model, whether in the UC of Nursing in Women’s Health, or in the UC of Nursing in Elderly Health or other”* (PTE_3); *“At the level of the maternal and obstetric health specialty, yes!”* (PTS_3).

There are universities that do not address sex education, but professors consider it to be important, in these places it may still not be valued. *“I do not think it is valued enough to be part of the curriculum, but it should be. In the nursing course I think it should be a compulsory subject”* (PTE_6, PTE_9, IT_4).

### 3.4. Contents

Regarding to the content theme, we can say that it has several sub-themes (1) gender issues; (2) ethical dimension of sexuality; (3) Contraceptive methods and sexually transmitted infections; (4) Violence; (5) Emotions and affections; (6) Body Image and female genital mutilation.

In Italy and Portugal the issue of gender-based violence is worked on adults and children (IT_1, IT_3, PTE_4, PTE_5), “*I try to sensitize students on the topic, not only because they are involved in the assessment, but also to raise awareness of what can be premonitory signs, being a spokesperson for the peer group”* (IT3); *“this school has several important works, works associated with the Ministry of Education that are related to gender identity issues, so it is a school that is very used to dealing with these themes in which there is also an intersection with sex education”*(PTS_10).

The ethical dimension of sexuality is reported by a couple of interviewees: an Italian teacher states that ethics “*concerns the dimension of modesty and respect for the dignity of the other*” (IT_3). From the University of Evora, a reflection is made on the awareness of the student in this regard: “*I make students aware of aspects that are not directly related to sexuality, but are sexuality,* e.g.*, when we ask for the patient’s consent for hygiene care (hygiene of the genital organs)*” (PTE_5). In considering the experience of the person, reference is made to the patient, the professional (IT_2), but also the issue of gender equality (PTS_10); “*What we have now seen with some regularity and more often are, for example, (…) couples of 2 women who show up to have children, for example, which is an issue that is starting to be seen nowadays more often and that requires openness and respect on our part like any other type of couple and especially when we have students we try to instill in them this way of dealing with people who have to be the same as everyone else respect the sexuality of all people and provide the best possible care in any of the situations*” (PTS_2).

Sexually transmitted diseases are covered in depth in terms of diagnosis, prevention, and treatment by all the universities involved, in the same way as pregnancy and contraception. *“Other aspects of sexuality… STIs, unwanted pregnancy and contraception”* (PTS_4); *“… methods of contraception (…) I think especially for 2 years and more on sexual and reproductive health, infectious diseases”* (PTE_9); “I teach Sexually Transmitted Diseases, prevention practices, diagnosis and treatment of STDs” (IT_10, ES_4).

Addressing topics such as domestic violence and dating violence*.”…because unfortunately there are, to mistreatment, so I do this about this(…) also the sexual injury that can be done and how to stem it, on the child depending on the age and which communication strategies maybe to use,(…) in gynecology, where there is often a direct access where there are abuses and therefore we enter this aspect, which is always part of this sexual sphere where you go to harm the person”* (IT_1); “(...) *we talk about violence in the relationship and address the various forms of sexual violence.*” (PTE_4; PTE_5).

The part concerning the student’s emotions at stake is reported under several headings: “*Students address the subject related to emotional caresses*” (PTE_3); and “*We talk about principles, values, and affections, before addressing sexuality itself*” (PTE_4); *“I know that other psychology colleagues addressed issues related to values, attitudes, including values and attitudes associated with sex education and also gender equality.”* (PTS_10).

Some interviewees refer to the cultural aspect of sexuality concerning anthropology (IT_6, IT_7, PTE_4), with specifics related to female genital mutilation (IT_3, PTS_4). *“Female genital mutilation that we thought was important we integrated into our curricular unit (…) it cannot be just for maternal and obstetric health nurses; it has to be for all nurses because mutilation is not identified only in the delivery room”* (PTS_4). About body image, it is addressed in nursing of surgical specialties, in the case of mastectomies, hysterectomies, etc. *“(…) example in the disturbance of the body image, citing a woman in a mastectomy for example, the importance of the reconstruction, however, the restoration of the body image also from a personal point of view but also from a relationship point of view”* (IT_4); *“When let us go to the surgery area, for example, one of the dimensions that I remember, is the care for people with ostomies and who have a colostomy bag, in the dimension of the preparation for the return home that is done from the first day, the questions of sexuality must be addressed with the couple and worked on effectively. While the professional must work on the issues of skin care, he must have the same type of care appreciation in all dimensions. This care must be integrated; it cannot be torn apart. The person’s experience of sexuality is part of their life; it cannot be a separate drawer* ***”*** (PTS_1).

### 3.5. Barriers

Although many teachers report that they have no problems in dealing with the topic (IT_5, IT_6, IT_8, IT_10, ES_3, ES_5, ES_8, ES_9, ES_10, PTE_1, PTE_9, PTE_10), awareness of the possible barriers is many and common to all countries. The first, in chronological order, related to the first level of education, that is, the school path before university, which should introduce the topic (IT_3, IT_4, PTE_6, PTS_3), starting with body awareness (ES_5). Dealing with the topic at school, in addition to how it is dealt with in the family, brings added value: “*school speaks in technical terms (...) it has a pedagogical organization”* (PTS_11) and *“at the level of civic training*” (PTS_9). It is widely recognized that it is more difficult to deal with adolescents than with nursing students (PTE_4).

More generally, on a socio-cultural level, sexuality is still referred to as a (2) taboo subject: “*in our reality, there is still a taboo on sexuality in general*” (IT_1) and this is reported by all countries (IT_5, ES_1, ES_6, PTS_3, PTS_3, PTS_4, PTS_8, PTS_9). “*Sometimes we ask a question, but we perfectly feel that the person is ashamed or does not want to talk*” (PTS_3); “*In the case of health professionals, I think that a barrier they may encounter when addressing the issue may be that it is considered a taboo subject, and that is why clients/patients do not open up the issue”* (PTE_3); *“(Difficulty?) Yes, I believe in those somewhat conventional paradigms of the taboo, because in our reality there is always a taboo, sexuality in general. (…) perhaps the taboo, the grinning happens every now and then* “(IT_1);

*“Break taboos with sex in the climacteric and old age”* (ES_6).

Among the barriers related to (3) students’ knowledge and reactions, the primary barrier is related to the difficulty in tackling the topic (IT_1, IT_3, PTE_1, PTE_2, PTS_5, PTS_9), especially about the age of the target population (PTS_5, PTE_6). While awareness and openness to different orientations of sex are recognized (PTS_11), it remains difficult to address the specific issues: “*often young people go to seek knowledge in their relational groups in schools (…) or with “Dr. Google” (…), subject to bad information (…), to deviations that are not healthy*” (PTS_11, PTS_8); “*Sometimes the fact that the student has low participation can be a barrier, because the student is afraid of sharing more intimate matters, they are afraid of bullying, of cyberbullying, so we have to be sensitive and accept the difficulties of the students.”* (PTE_4); *“It is difficult to capture the attention of the students”* (ES_7). In general, there is great difficulty in sharing issues related to sexuality, even if they are related to traineeship experiences (PTE_4, PTE_5).

Also, in the approach to sexuality in clinical training contexts, the difficulty emerges related to the sense of modesty and the difficulty of touching the body of the person, who is often elderly (IT_2). If the topic is introduced in the first year, without a direct correlation or experience with clinical practice, the student risks not understanding its meaning (PTS_1). *“Some of the dimensions of sexuality are worked on, for example, in the first year, in Fundamentals of Nursing I... they are part of the program, it is not always easy, students later understand how these areas are important aspects to be integrated later...”* (PTS_1).

Cultural and religious issues (4) are also a barrier identified in sexuality education. The cultural and religious diversity of patients to whom the nurse provides care, influences their sexual practice. *“But I would say that, as I said before, it is really a question, precisely cultural, where the awareness of the potential of sexuality has not yet matured(...) we need to free ourselves from our point of view, I would say cultural in relation to traditions”* (IT_3); *“We currently have an added difficulty, which is cultural issues, I have many migrants in my workplace. Issues of contraception, for example, must be addressed across cultures and very differently”* (PTS_3); *“It can come from an environment, or from a religion, from a paternalistic family, which does not facilitate the conversation about sexuality.”* (PTE_5).

The (5) lack of time to address sexuality education is mentioned in the four countries (IT_1, IT_3, IT_7, IT_9, IT_10, ES_2; ES_4, PTS_1, PTS_2, PTS_3, PTE_ 3, PTE_8). *“There is not much time to develop this topic, since the subjects have a very concentrated”* (ES_4); *“The time usually ends up being more limited for these types of approaches and for these types of issues that often pass a little to the side”* (PTS_2); *“…during clinical teaching, not only the subject of sexual education is addressed. So many more topics are covered, and I end up not having time to explore sex education and the importance of teaching sex education.”* (PTE_8). Despite this lack of time, one respondent reports: “*Time is something that we do not have enough of, but I usually say that when we want it and when it becomes important, if we feel that it is very important to address this issue, I think that everyone can find a curriculum to start addressing these issues*” (PTE_10).

The professional aspects (6) related to the education and comfort of professionals are also described as a barrier. *“Sometimes they are not awake to address issues related mainly to these new complexities, that revolve around sexuality and that are more in evidence today, the issue, for example, of non-binary, binary people, all these new concepts that arise today and that I believe that many health professionals and many people in education do not have knowledge or sometimes do not feel comfortable addressing these issues”* (PTS_2); “*The teacher has to feel comfortable with this theme, he has to say, he has to prepare himself adequately to teach this theme. (...) They need to equip themselves with tools and strategies that allow them to connect to the student.”* (PTE_1). The issue of the barrier related to the sex/gender of the teacher appears less frequently but is mentioned by more respondents (IT_2, PTS_2): **“***the fact that nursing is mainly female students, teachers, especially male teachers, may have difficulties in dealing with certain questions from these young women*” (PTE_5). An interview refers to the issue from a gender diversity perspective: “(…) *now in the approach of new typologies, (…) everything that is linked to gays, lesbians, transgender (…) the type of language that must be used, the acceptance of these situations. (...) there are health professionals who still do not understand these new typologies well (…) there are colleagues [pediatric nurses] who often use political correctness (…), they feel embarrassed to talk about these issues*” (PTS_11).

There are interviewees who do not identify any barriers in approaching sexuality education (IT_5, IT_6, ES_3, ES_5, PTE_1). *“I do not find it difficult to teach sexual education”* (ES3); *“So far, I have not identified any, it is easy to work on this topic with students of this educational level (undergraduate)* (PTE_1).

### 3.6. Teachers’ attitudes

The vast majority of respondents report (1)feeling comfortable dealing with the topic (IT_9, IT_10, ES_5, ES_6, ES_7, ES_8, ES_9, PTE_3, PTE_4, PTE_6, PTE_7, PTE_11, PTE_12, PTS_1, PTS_2, PTS_6, PTS_7, PTS_8, PTS_9, PTS_10): “*I consider that I have bases to help in this orientation, I happen to deal well with this subject, I have no problem, I have no shame, it is an area that I enjoy working*” (PTS_3). One lecturer reported promoting classroom discussion and debate with students (PTS_10). Some lecturers link this to the fact that they treat the topic by relating it to specific clinical contexts (IT_1, IT_4, IT_7, PTE_3, PTE_5, PTS_1, PTS_9).

Some state that they have no difficulties, although they have never dealt with the topic, and declare themselves open to this possibility (IT_5, ES_2, ES_4, ES_10, ES_11, ES_12). Some lecturers correlate this with specific training and years of experience (IT_7, PTE_8, PTE_9, PTS_2) or state lack of and need for specific training (IT_8, IT_9); one lecturer correlates this element with the fact that he is a white heterosexual male (IT_6), contrary to an interview reported earlier.

One of the elements reported as facilitating is the introduction of the topic from a specific request/problem of the students (IT_2), also with an accompanying approach to the growth of future professionals (IT_3).

One teacher report: “*It is not so difficult to work on sexual health issues with students, it is often difficult to try to pass on to students what was difficult for me, to answer without my judgment of value being present and which helped me to deconstruct and arrive to what I am today* (PTS_1).

The aspects reported as (2) discomfort refers to aspects already dealt with: the lack of correlation to the clinical care (IT_1, IT_2), lack of specific teacher training (PTS_6), the age of the teacher (very distant from students) (PTS_6), or topics related to individual affectivity (IT_4). *“I personally would find it difficult to talk about the more psychological aspects or those related to affectivity, personal experience, rather than difficulties of a sexual or relationship nature”* (IT_4); *“Maybe I need to deepen some knowledge, I will not say no. Even in the way of approaching them, because it is a difficult topic to approach (…) Personally, I think it is a difficult topic for some professors to address… the older ones and for the students”* (PTS_6).

### 3.7. Teacher training

In the topic of teacher training, some of the elements already mentioned above emerge. Some teachers share the need for a (1) comparison between colleagues, within their university (IT_2, PTS_4), to follow “*a common thread in dealing with this type of topic in the three years of the course, also in the awareness that sexual health has many aspects*” (IT_2).

More generally, teachers perceive the need to involve other professionals with (2) specific training or expertise (IT_2, IT_3, IT_4, IT_5, IT_6, ES_1, ES_2, ES_3, ES_4, ES_5, ES_6, ES_7, ES_8, ES_9, ES_10, ES_11, ES_12, PTE_2, PTE_7, PTS_3, PTS_5, PTS_6, PTS_7, PTS_9, PTS_10), in order not to trivialize the meaning of training (IT_4). “*I do not think I would already be willing to give. First, I must educate myself and know what I can talk about, then, based on that, I can go on to the subject in question. Now, I do not feel ready* (…)” (PTE_10).

This expertise could be clinical (e.g., nurses working in dedicated services, midwives) (IT_1, IT_4, IT_5, ES_2, ES_3, ES_4, ES_5, ES_8, ES_10, ES_12, PTE_12, PTS_9, PTS_10), communicative (PTS_3), intercultural/anthropological (IT_3, ES_5, PTS_11), psychological and sociological (IT_6), about public health (IT_5) or human sexuality (Level II training) (PTS_8, PTS_9, PTS_11): “…*this topic was taught by a professor with a doctorate in particular on human sexuality and that was a great asset because she had a good body of specific knowledge (…). I believe that it is an asset, if possible, for the people who teach to be close or connected to this area*” (PTS_8). About the communication, one teacher reported: “*Nurses must be prepared, able to have appropriate language, so that the couple feels free to clarify any doubts they have about sexuality”* (PTS_4). A broader look at the topic suggests: “*now we are expanding more because of the goals of sustainable development, there are also more topics that future teachers have to receive in their training*” (PTS_10).

One aspect reported is the (3) importance of continuous updating of knowledge, both subject-specific (IT_5, IT_9, PTS_9, PTS_11) and pedagogical strategies (IT_9, ES_5, ES_12, PTS_9, PTS_11): “*Even the approaches themselves tend to evolve very fast (...) one is up to date (...) a person who is used to doing research on sexuality (…) with a vision more scientific about the situation*” (PTS_11). “(…) *Is a subject that is constantly evolving, constantly changing and we have to learn new techniques and develop new skills*” (PTE_8).

While recognizing the need for collaboration, one teacher reflects on the risk of compartmentalization of nursing (IT_2). To this reflection, he/she adds: “*it is a dimension that is multifactorial, so I think it requires the integration of different knowledge aspects from different disciplines, precisely also because of the delicacy of the thing. (...) it does not detract from the importance of specific teaching and training which, also because of the area related to a series of taboos, cultures,* etc.*, certainly calls for a multiple knowledge approach”* (IT_3). Some teachers report feeling prepared on the contents but having difficulties with the pedagogical strategies suggested to better deal with the complexity and delicacy of the topic (ES_2, ES_3, ES_4).

## 4. Discussion

The importance of the inclusion of sexuality in nursing curricula is beyond dispute [World Health Organization (WHO), n.d.]; all the participants in the study agreed on this aspect as an identity component of the person and therefore indispensable for holistic care. Despite this certainty, many nurses do not feel comfortable with sexuality (Martel et al., 2017) and the main risk of this aspect is that the dimension is not addressed in the clinical setting (Moore et al., 2013; Krouwel et al., 2015; Jonsdottir et al., 2016).

The study shows that nursing education devotes little attention to sexuality, which is dealt with in specific moments, within individual teachings, and that teachers do not always have an overview.

The elements developed and reported are diverse and refer to different aspects, not only clinical care. Among the general topics are the meanings of sexuality and, considering them with a double sociological and clinical valence, cultural specifics, and gender-based violence. Among the more health care specific topics, we find gender medicine, sexually transmitted diseases (Buston et al., 2002), female genital mutilation, contraceptive methods (Buston et al., 2002), body image disturbance, and ethical assistance aspects that are part of needed sexuality education. In general, no specific disciplines emerge, but a wide variability of topics becomes part of individual teaching modules, without any real program coordination (Roberto et al., 2007), and which remains managed by the individual teacher. Although the added value of the cross-curricular subject matter is repeatedly reported, the risk of compartmentalization of knowledge is highlighted. Several studies in the literature emphasize the importance of an increased focus on sexuality, as it not only creates knowledge and skills but also helps professionals feel more comfortable dealing with such a complex and sensitive topic (Kirby et al., 2007; Coleman et al., 2013; Baggio et al., 2015;Turner et al., 2016; Beebe et al., 2021).

The barriers that emerged from the study are diverse and attributable to students, lecturers, and the institution. Added to these is the sociocultural barrier that struggles to eliminate the conception of the subject as forbidden, despite a great openness in recent years on the part of the younger generation. In this regard, because sex and gender diversity is an abundantly debated and daily topic, especially in the media, there remains the difficulty of considering sexuality to be important related to illness and the elderly person, especially among the young students in our nursing education programs.

The main barrier related to the teacher appears to be the lack of specific training, both about content and teaching strategies, as already reported in the literature (Ninomiya, 2010; Cohen et al., 2012; Mkumbo, 2012; Klaeson et al., 2017; Rose et al., 2018). It is important to point out that these elements appear to be closely related to the teacher’s ease in dealing with the topic (Cohen et al., 2012). Although most of the respondent’s report feeling comfortable, it is necessary to underline the fact that part of the sample has never dealt with the topic, even if they declared themselves open to this possibility.

Among the institutional barriers, the lack of time appears to be the main one in all the countries involved in the study and is in line with the findings of the literature (Moore et al., 2013; Perez et al., 2013; Krouwel et al., 2015; Jonsdottir et al., 2016). This constraint should be seen because of what was previously noted, regarding the lack of general coordination at the curriculum and institutional level (Perez et al., 2013).

The study has limitations because it is a subjective approach, typical of qualitative studies, which makes it impossible to generalize its results. However, its contribution lies in the possibility of encouraging reflection among nursing professors on sexuality education, in order to create strategies that allow transforming the identified barriers into an opportunity to improve the quality of teaching on this topic.

## 5. Conclusion

Nursing professors consider sexuality education to be very important. In the vast majority, this topic is integrated into the study plan of the nursing course where they teach. The same can be integrated into specific disciplines, such as child health and pediatrics nursing, maternal and obstetric health nursing, psychology, etc., or it can be approached transversally in different curricular units, through the implementation of a Theoretical model, for example, with Nancy Roper’s Theoretical Model, integrates sexuality education into the life activity “expressing sexuality.” The most discussed contents within this theme are gender issues, ethical and cultural issues, sexually transmitted infections, violence, emotions and affections, alteration of body image and female genital mutilation.

The common barriers that teachers encounter when teaching content about sexuality are the students’ education level, it being a taboo subject, students’ knowledge and reactions to the topic, religious and cultural issues, time available to talk about the topic and related professional aspects. The subject, namely training on the subject and the comfort of professionals. Despite these issues, teachers report that they feel comfortable approaching the topic of sexuality.

Teachers understand that to teach this theme, there must be a standardization of the way in which the content is taught. Teachers who teach sexuality must have specific training or specialization in the area, and they need a continuous updating of knowledge, either in relation to the specific content, or in relation to the pedagogical strategies.

## Data availability statement

The original contributions presented in the study are included in the article/supplementary material, further inquiries can be directed to the corresponding author.

## Ethics statement

The studies involving human participants were reviewed and approved by Research Ethics of University Castilla-La Mancha (UCLM). CAU-661803-V4Z4. The patients/participants provided their written informed consent to participate in this study.

## Author contributions

CG and DM: conceptualization. EC and FF: data curation. MB-R and SP: formal analysis. SG-C: funding acquisition. ML and CG: investigation. FF and MB-R: methodology and validation. DM and SG-C: project administration and visualization. ML and EC: resources. CG: software. SG-C and SP: supervision. MB-R and SG-C: writing—review and editing. All authors have read and agreed to the published version of the manuscript.

## Funding

This article is part of the European Project: Educating in Sexuality Advancing European Health (EdSeX). Subsidized by the Ministry of Universities (SEPIE), and by the European Union under the code: 2021-1ES01-KA220-HED-000023306. Italy. The funders had no role in study design, data collection and analysis, decision to publish or preparation of the manuscript.

## Conflict of interest

The authors declare that the research was conducted in the absence of any commercial or financial relationships that could be construed as a potential conflict of interest.

The reviewer AC-O declared a shared affiliation with the author SG-C to the handling editor at the time of review.

## Publisher’s note

All claims expressed in this article are solely those of the authors and do not necessarily represent those of their affiliated organizations, or those of the publisher, the editors and the reviewers. Any product that may be evaluated in this article, or claim that may be made by its manufacturer, is not guaranteed or endorsed by the publisher.

## References

[ref1] Baggio G. (2015). Dalla medicina di genere alla medicina genere-specific. Ital. J. Gend. Spec. Med. 1, 3–5. doi: 10.1723/2012.21900

[ref2] Beebe S. Payne N. Posid T. Diab D. Horning P. Scimeca A.. (2021). The lack of sexual health education in medical training leaves students and residents feeling unprepared. J. Sex. Med. 18, 1998–2004. doi: 10.1016/J.JSXM.2021.09.011, PMID: 34711518

[ref3] Brancaleoni A. P. Oliveira R. R. Franciscati da Silva C. M. (2018). Sexual education and university: undergraduates’ understanding of sexuality and gender. Rev. Bras. Ensino Superior 4, 25–42. doi: 10.18256/2447-3944.2018.v4i4.2563

[ref4] Braun V. Clarke V. (2019). Reflecting on reflexive thematic analysis. Qual. Res. Sport Exerc. Health 11, 589–597. doi: 10.1080/2159676X.2019.1628806

[ref5] Buston K. Wight D. Hart G. Scott S. (2002). Implementation of a teacher-delivered sex education program: obstacles and facilitating factors. Health Educ. Res. 17, 59–72. doi: 10.1093/HER/17.1.59, PMID: 11888044

[ref6] Chen E. Barrington C. (2017). “You can do it anywhere”: student and teacher perceptions of an online sexuality education intervention. Am. J. Sex Educ. 12, 105–119. doi: 10.1080/15546128.2017.1298066

[ref7] Cohen J. N. Byers E. S. Sears H. A. (2012). Factors affecting Canadian teachers’ willingness to teach sexual health education. Sex Educat. 12, 1–18. doi: 10.1080/14681811.2011.615606

[ref8] Coleman E. Elders J. Satcher D. Shindel A. Parish S. Kenagy G.. (2013). Summit on medical school education in sexual health: report of an expert consultation. J. Sex Med. 10, 924–938. doi: 10.1111/JSM.12142, PMID: 23551542

[ref9] Eisenberg M. E. Madsen N. Oliphant J. A. Resnick M. (2012). Policies, principals and parents: multilevel challenges and supports in teaching sexuality education. Sex Educat. 12, 1–13. doi: 10.1080/14681811.2011.615614

[ref10] Evans A. E. Edmundson-Drane E. W. Harris K. K. (2000). Computer-assisted instruction: an effective instructional method for HIV prevention education? J. Adolesc. Health 26, 244–251. doi: 10.1016/S1054-139X(99)00093-2 10734271

[ref11] Huang C. Y. Tsai L. Y. Tseng T. H. Li C. R. Lee S. (2013). Nursing students’ attitudes towards provision of sexual health care in clinical practice. J. Clin. Nurs. 22, 3577–3586. doi: 10.1111/JOCN.12204, PMID: 23651413

[ref12] Jonsdottir J. I. Zoëga S. Saevarsdottir T. Sverrisdottir A. Thorsdottir T. Einarsson G. V.. (2016). Changes in attitudes, practices, and barriers among oncology health care professionals regarding sexual health care: outcomes from a 2-year educational intervention at a University Hospital. Eur. J. Oncol. Nurs. 21, 24–30. doi: 10.1016/J.EJON.2015.12.004, PMID: 26952675

[ref13] Kirby D. Laris B. A. Rolleri L. (2006). Impact of sex and HIV education programs on sexual behaviors of youth in developing and developed countries. USA: Family Health International

[ref14] Kirby D. B. Laris B. A. Rolleri L. A. (2007). Sex and HIV education programs: their impact on sexual behaviors of young people throughout the world. J. Adolesc. Health 40, 206–217. doi: 10.1016/J.JADOHEALTH.2006.11.143, PMID: 17321420

[ref15] Klaeson K. Hovlin L. Guvå H. Kjellsdotter A. (2017). Sexual health in primary health care - a qualitative study of nurses’ experiences. J. Clin. Nurs. 26, 1545–1554. doi: 10.1111/JOCN.13454 27324221

[ref16] Krouwel E. M. Nicolai M. P. J. Van Steijn-Van Tol A. Q. M. J. Putter H. Osanto S. Pelger R. C. M.. (2015). Addressing changed sexual functioning in cancer patients: a cross-sectional survey among Dutch oncology nurses. Eur. J. Oncol. Nurs. 19, 707–715. doi: 10.1016/J.EJON.2015.05.005, PMID: 26051072

[ref17] Martel R. Crawford R. Riden H. (2017). “By the way…. how’s your sex life?” A descriptive study reporting primary health care registered nurses’ engagement with youth about sexual health. J. Prim. Health Care 9, 22–28. doi: 10.1071/HC17013, PMID: 29530184

[ref18] Mkumbo K. A. (2012). Teachers’ attitudes towards and comfort about teaching school-based sexuality education in urban and rural Tanzania. Glob. J. Health Sci. 4, 149–158. doi: 10.5539/GJHS.V4N4P149, PMID: 22980351 PMC4776935

[ref19] Moore A. Higgins A. Sharek D. (2013). Barriers and facilitators for oncology nurses discussing sexual issues with men diagnosed with testicular cancer. Eur. J. Oncol. Nurs. 17, 416–422. doi: 10.1016/J.EJON.2012.11.008, PMID: 23290540

[ref20] Mueller T. E. Gavin L. E. Kulkarni A. (2008). The association between sex education and youth’s engagement in sexual intercourse, age at first intercourse, and birth control use at first sex. J. Adolesc. Health 42, 89–96. doi: 10.1016/J.JADOHEALTH.2007.08.002, PMID: 18155035

[ref21] Ninomiya M. M. (2010). Sexual health education in Newfoundland and Labrador schools: junior high school teachers’ experiences, coverage of topics, comfort levels and views about professional practice. Can. J. Hum. Sex. 19, 15–26.

[ref22] Perez M. A. Luquis R. Allison L. (2013). Instrument development for measuring teachers’ attitudes and comfort in teaching human sexuality. Am. J. Sex Educ. 35, 24–29. doi: 10.1080/19325037.2004.10603601

[ref23] Roberto A. J. Zimmerman R. S. Carlyle K. E. Abner E. L. (2007). A computer-based approach to preventing pregnancy, STD, and HIV in rural adolescents. J. Health Commun. 12, 53–76. doi: 10.1080/10810730601096622 17365349

[ref24] Rose I. D. Boyce L. Murray C. C. Lesesne C. A. Szucs L. E. Rasberry C. N.. (2018). Key factors influencing comfort in delivering and receiving sexual health education: middle school student and teacher perspectives. Am. J. Sex Educ. 14, 466–489. doi: 10.1080/15546128.2019.1626311, PMID: 33897308 PMC8064695

[ref25] Sung S. C. Jiang H. H. Chen R. R. Chao J. K. (2016). Bridging the gap in sexual healthcare in nursing practice: implementing a sexual healthcare training program to improve outcomes. J. Clin. Nurs. 25, 2989–3000. doi: 10.1111/JOCN.13441, PMID: 27324599

[ref26] Turner D. Nieder T. O. Dekker A. Martyniuk U. Herrmann L. Briken P. (2016). Are medical students interested in sexual health education? A nationwide survey. Int. J. Impot. Res. 28, 172–175. doi: 10.1038/IJIR.2016.25, PMID: 27225710

[ref27] United Nations (2030). Transforming our world: the 2030 Agenda for sustainable development. https://sustainabledevelopment.un.org/content/documents/21252030%20Agenda%20for%20Sustainable%20Development%20web.pdf (Accessed May 19, 2023).

[ref28] Vamos S. Zhou M. (2003). Educator preparedness to teach health education in British Columbia. Am. J. Health Educ. 38, 284–292. doi: 10.1080/19325037.2007.10598983

[ref29] Vilaça T. (2017). Agenda 2030 na educação em ciências e educação em sexualidade: implicações na formação docente

[ref30] World Health Organization (WHO). (n.d.) Sexual and reproductive health and research (SRH). Available at: https://www.who.int/teams/sexual-and-reproductive-health-and-research/key-areas-of-work/sexual-health/defining-sexual-health (Accessed January 3, 2023).

[ref31] Yingling C. T. Cotler K. Hughes T. L. (2017). Building nurses’ capacity to address health inequities: incorporating lesbian, gay, bisexual and transgender health content in a family nurse practitioner programme. J. Clin. Nurs. 26, 2807–2817. doi: 10.1111/JOCN.13707, PMID: 28029727

